# Time-Course of Neuromuscular Changes during and after Maximal Eccentric Contractions

**DOI:** 10.3389/fphys.2016.00137

**Published:** 2016-04-18

**Authors:** Valentin Doguet, Marc Jubeau, Sylvain Dorel, Antoine Couturier, Lilian Lacourpaille, Arnaud Guével, Gaël Guilhem

**Affiliations:** ^1^Laboratory “Movement, Interactions, Performance” (EA 4334), Faculty of Sport Sciences, University of NantesNantes, France; ^2^Laboratory Sport, Expertise and Performance (EA 7370), Research Department, French Institute of Sport (INSEP)Paris, France

**Keywords:** central activation ratio, voluntary activation level, evoked torque, plantar flexors, muscle damage

## Abstract

This study tested the relationship between the magnitude of muscle damage and both central and peripheral modulations during and after eccentric contractions of plantar flexors. Eleven participants performed 10 sets of 30 maximal eccentric contractions of the plantar flexors at 45°·s^−1^. Maximal voluntary torque, evoked torque (peripheral component) and voluntary activation (central component) were assessed before, during, immediately after (POST) and 48 h after (48 h) the eccentric exercise. Voluntary eccentric torque progressively decreased (up to −36%) concomitantly to a significant alteration of evoked torque (up to −34%) and voluntary activation (up to −13%) during the exercise. Voluntary isometric torque (−48 ± 7%), evoked torque (−41 ± 14%) and voluntary activation (−13 ± 11%) decreased at POST, but only voluntary isometric torque (−19 ± 6%) and evoked torque (−10 ± 18%) remained depressed at 48 h. Neither changes in voluntary activation nor evoked torque during the exercise were related to the magnitude of muscle damage markers, but the evoked torque decrement at 48 h was significantly correlated with the changes in voluntary activation (*r* = −0.71) and evoked torque (*r* = 0.77) at POST. Our findings show that neuromuscular responses observed during eccentric contractions were not associated with muscle damage. Conversely, central and peripheral impairments observed immediately after the exercise reflect the long-lasting reduction in force-generating capacity.

## Introduction

Eccentric contraction represents a motor action whereby the muscle-tendon unit is forcibly lengthened while the muscle is activated. Such contractions have been recognized to generate greater torque than isometric or concentric modes (Kellis and Baltzopoulos, 1995), thanks to a large contribution of passive structures (Herzog, 2014). In parallel, compared with concentric and isometric contractions, lower surface electromyographic (EMG) activity and voluntary activation have been reported during eccentric contractions (Westing et al., 1991; Babault et al., 2001). This non-maximal motor unit recruitment showed evidences of an unique neural strategy during eccentric contractions (Enoka, 1996), arising from spinal (e.g., facilitation of pre- and post-synaptic inhibitions), and supra-spinal (e.g., cortical excitability enhancement) components (see Duchateau and Baudry, 2014 for a review). Further, it is well-established that unaccustomed or repetitive eccentric contractions result in structural and histochemical disruptions in the activated muscle, referred to as muscle damage (Armstrong, 1984; Mair et al., 1992). Whilst functional (e.g., drop in muscle strength, muscle soreness) and physiological (e.g., muscle fiber disruptions, inflammatory processes) events associated with muscle damage have been well-described (Mair et al., 1992; Faulkner et al., 1993; Paulsen et al., 2012), neuromuscular causes of exercise-induced muscle damage remain not fully understood.

Previous studies theorized that the lower muscle activation observed during eccentric contractions, compared with the other contraction modes, could generate high tension levels in a reduced active muscle fibers population, favoring mechanical disturbances (Newham et al., 1983; Moritani et al., 1987). Conversely, other authors argued that this incomplete muscle activation could act as a tension-limiting phenomenon protecting against further muscle damage (Westing et al., 1991). Research focused on the repeated bout effect phenomenon, which refers to the protection conferred from an initial eccentric exercise to a second exercise bout, demonstrated the influence of neural factors on the magnitude of muscle damage (McHugh, 2003). For instance, Warren et al. (2000) reported a shift in the EMG frequency content toward a low frequency band in the second bout of eccentric exercise, associated with a reduction in indirect markers of muscle damage (e.g., drop in muscle strength, muscle soreness). These authors suggested that slow-twitch fibers, which are more resilient to muscle damage (Fridén et al., 1983), were preferentially recruited by the central nervous system during the second bout. In addition, other studies showed that an initial eccentric exercise bout of elbow flexors could reduce the muscle damage symptoms of both ipsilateral and contralateral arms (Howatson and van Someren, 2007; Starbuck and Eston, 2012), also suggesting an influence of neural factors on the magnitude of muscle damage. In contrast, Hubal et al. (2007) reported that changes in torque generating capacity after eccentric contractions of elbow flexors were associated with peripheral changes characterized by evoked torque, but not with neural changes measured using both EMG and voluntary activation. Thus, this study suggested that neural factors did not influence eccentric exercise-induced muscle damage. However, the relationship between the central nervous system and eccentric exercise-induced muscle damage has mainly been investigated on the basis of the delayed responses to damaging exercises (Prasartwuth et al., 2005; Hubal et al., 2007; Racinais et al., 2008). Thus, these findings allowed to assess some origins of the force impairments resulting from damaging exercise, without fully explaining the causal mechanisms responsible for muscle damage. As far as can be ascertained, an assessment of central (i.e., central nervous system) and peripheral (i.e., muscle) properties during eccentric contractions has never been explored.

The present study evaluated voluntary activation and evoked torque as indexes of central and peripheral components, respectively, during and after (immediately and 48 h post-exercise) isokinetic eccentric contractions of plantar flexors. Based on previous evidences of a neural influence on muscle damage through repeated bout effect phenomenon (Warren et al., 2000; Howatson and van Someren, 2007), it was hypothesized that central function assessed during eccentric contraction was related to muscle damage markers. Moreover, peripheral changes during eccentric contractions were expected to be closely related to muscle damage symptoms as muscle function has been suggested to represent the main part of alterations associated with muscle damage (Warren et al., 2002).

## Materials and methods

### Participants

Eleven healthy young subjects (5 males, 6 females) with no history of neurological or neuromuscular diseases, and not practicing lower limbs resistance training, participated in this study (age: 25.9 ± 3.9 years; height: 173.7 ± 9.9 cm; weight: 68.7 ± 11.4 kg). All participants gave their written informed consent after they have been informed about the experimental procedures and possible risks and discomfort. This study was approved by the local ethics committee and conducted according to the Declaration of Helsinki (2004).

### Experimental design

A cross-sectional study design has been used in the present investigation. Participants attended to three separate sessions. A first test session was dedicated to the assessment of baseline measurements. Two days later, participants performed isokinetic eccentric contractions (i.e., eccentric exercise, section Eccentric Exercise) and the same tests as the initial test session were repeated immediately (POST) and 2 days (48 h) after the exercise.

### Test sessions

#### Voluntary isometric peak torque

Two 3-s isometric maximal voluntary contractions (MVC) of plantar flexors were performed at 90° of ankle joint angle (i.e., foot perpendicular to the tibia), within a 1-min rest period, except at POST where only one trial was achieved to minimize the time elapsed after the exercise. Participants were lying prone (hip angle: ~0°; knee angle: ~0°) on a Con-Trex MJ isokinetic dynamometer (CMV AG, Dübendorf, Switzerland) with the right ankle fixed to the dynamometer's attachment with non-compliant straps. A trunk harness system was also attached to the ergometer structure so that the body did not move on the platform. Torque and position signals were digitized by a 12-bit analog to digital converter (DT 9804, Data Translation, Marlboro, USA) and sampled at 5000 Hz.

#### Neuromuscular tests

**Figure 1:**
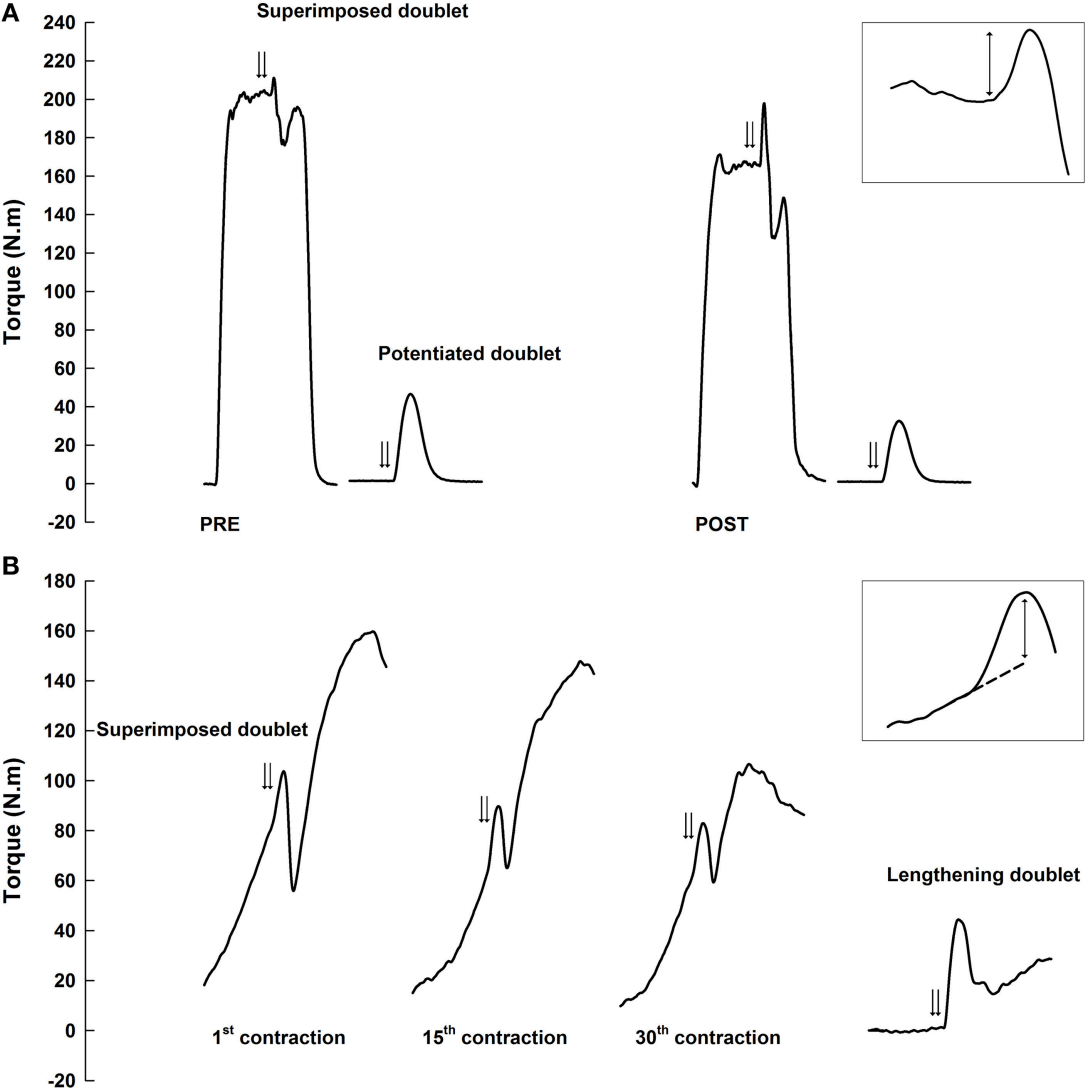
**Example of torque traces for maximal voluntary isometric contractions before and immediately after the exercise (A) and for maximal voluntary eccentric contractions during the exercise (B)**. Double arrows represent paired stimulations superimposed to contractions (superimposed doublet) and evoked on the resting muscle after isometric contractions (potentiated doublet) or during a passive dorsiflexion cycle (lengthening doublet). Superimposed doublets are enlarged in the top right corner of each panel. Two-way arrows correspond to superimposed torques between the evoked torque and voluntary isometric peak torque **(A)** or extrapolated eccentric torque **(B)**.

For each MVC, (i) a supramaximal doublet was superimposed to the contraction (superimposed doublet; Figure 1A), over the isometric plateau and (ii) a supramaximal doublet was applied at rest, 2 s after the contraction (potentiated doublet). Rectangular paired pulses (duration: 1 ms, frequency: 100 Hz) were delivered by a constant-current Digitimer DS7A electrical stimulator (Digitimer Ltd., Hertfordshire, UK). The cathode (diameter = 1 cm; ADInstruments Pty. Ltd.) was positioned over the tibial nerve, in the popliteal fossa, and the anode (8 × 13 cm; Stimex, Rouffach, France) under the patella. A light pressure was applied on the cathode to stimulate closer to the nerve throughout the sessions. At the beginning of all sessions, the first intensity necessary to evoke the maximal amplitude of the twitch torque was determined using a 10-mA increment stimulation ramp. This intensity was increased by 30% for all following measurements.

#### Muscle soreness

Muscle soreness was assessed using a 100 mm visual analog scale with an anchor of “no pain” (0 mm) to “intolerable pain” (100 mm). Participants were asked to report the global perceived soreness level on the scale after the investigator palpated the following sites on the *triceps surae*: muscle insertions and muscle bellies of three plantar flexor muscles (i.e., *gastrocnemius medialis, gastrocnemius lateralis, soleus*).

### Eccentric exercise

Participants were lying prone on the isokinetic dynamometer and their ankle joint was passively moved (3 trials) from 90° to the maximal dorsiflexion angle they were able to achieve without discomfort. The greater angle reached in dorsiflexion (0°) was set as the limit of the range of motion and the starting position was set to obtain a whole constant range of motion of 60°. Thereafter, participants performed 10 sets of 30 maximal eccentric plantarflexions (2-min rest between sets), without preactivation period, at a constant angular velocity (45°·s^−1^). Participants were vigorously encouraged and the ergometer passively moved the foot to the starting position after each repetition at 45°·s^−1^. For each set, supramaximal doublets were software-driven superimposed to the first, 15th and last eccentric contractions when the foot crossed the 90° dynamometer's angle (i.e., foot perpendicular to the tibia; Figure 1B). Immediately after the last repetition of each set, a supramaximal lengthening doublet was applied at the same angle during a passive dorsiflexion cycle (45°·s^−1^). Torque signal was gravity-corrected (i.e., foot and accessory mass) throughout the overall range of motion with a 3rd degree polynomial function of torque measured during passive cycles (10°·s^−1^) performed over the same range of motion (Guilhem et al., 2010; Dello Iacono et al., 2016).

### Data processing

#### Test sessions

The voluntary isometric peak torque recorded during MVC was considered for all test sessions. For the considered MVC, the superimposed torque and potentiated torque evoked during and after isometric MVC, respectively, corresponded to the difference between torque before the pulse and the maximal evoked torque (Figure 1A). The superimposed torque was systematically corrected as follows to account for pulse delivered away from the actual maximal voluntary torque (Place et al., 2007):

(1) $$\begin{array}{l} ST_{corrected}=ST\times \frac{T_{stim}}{T_{\max}} \end{array}$$

where *ST*_*corrected*_ is the corrected superimposed torque; *ST* is the superimposed torque, *T*_*stim*_ is the torque at stimulation time, and *T*_*max*_ is the maximal measured torque.

Isometric voluntary activation was calculated using the voluntary activation level (*VAL*) method (Place et al., 2007) as follows:

(2) $$\begin{array}{l} VAL=\left[1-\frac{ST_{corrected}}{T_{potentiated}}\right]\times 100 \end{array}$$

where *VAL* is the voluntary activation level; *ST*_*corrected*_ is the corrected superimposed torque; and *T*_*potentiated*_ is the potentiated torque.

#### Eccentric exercise

Torque-angle relationship was measured set-by-set over the entire range of motion, excluding contractions with superimposed stimulations, and the mean value of the curve was reported as the mean voluntary torque. During the eccentric contractions, the superimposed torque was assessed by subtracting the maximal evoked torque to the torque that would have occurred without any stimulation at the same angle to the evoked torque (Babault et al., 2001). This “unstimulated” torque was quantified by linear extrapolation of the 50-ms torque preceding the stimulation (extrapolated torque; Figure 1B). Eccentric voluntary activation was calculated using the central activation ratio (CAR) method as follows:

(3) $$\begin{array}{l} CAR=\frac{T_{extrapolated}}{\left(T_{extrapolated}+ST_{corrected}\right)}\times 100 \end{array}$$

where *CAR* is the central activation ratio; *T*_*extrapolated*_ is the extrapolated torque; and *ST*_*corrected*_ is the corrected superimposed torque.

For passive cycles following all sets, the lengthening torque was assessed as the difference between torque before the lengthening doublet and the maximal evoked torque. Then, the lengthening torque was normalized to the baseline isometric potentiated torque.

### Statistical analysis

Outlier analyses were performed for all variables using the absolute deviation around the median (Leys et al., 2013), with a very conservative rejection criterion in accordance with Miller (1991). None of the participants was detected as outlier whatever the variable.

Statistical tests were performed using Statistica 7.0 software (Statsoft Inc., Tulsa, Oklahoma, USA). All data being normally distributed (Shapiro-Wilk test), variables measured during test sessions were compared between times using separated one-way repeated measures ANOVA. During the eccentric contractions, mean voluntary torque and lengthening torque were compared between sets using separated one-way repeated measures ANOVA. CAR was compared between sets and contractions (1st, 15th, and 30th) using two-way repeated measures ANOVA to observe intra- and inter-set changes. Since no contraction main effect was found, CAR was averaged between contractions for each set and compared between sets using one-way repeated measure ANOVA. *Post-hoc* analyses were performed when appropriate using a Newman-Keuls method. Partial eta square (_*p*_η^2^) values are reported as measures of effect size, with moderate and large effects considered for _*p*_η^2^ ≥ 0.07 and _*p*_η^2^ ≥ 0.14, respectively (Cohen, 1988). Separate linear Pearson's correlations (r) were performed to test for correlations between muscle damage markers at 48 h (i.e., voluntary peak torque and potentiated torque decrements) and neuromuscular parameters measured during (CAR and lengthening torque) and immediately after the exercise (relative changes for VAL and potentiated torque). The significance level was set at *p* < 0.05. Data are expressed as mean ± standard deviation (SD).

## Results

### Test sessions

**Figure 2:**
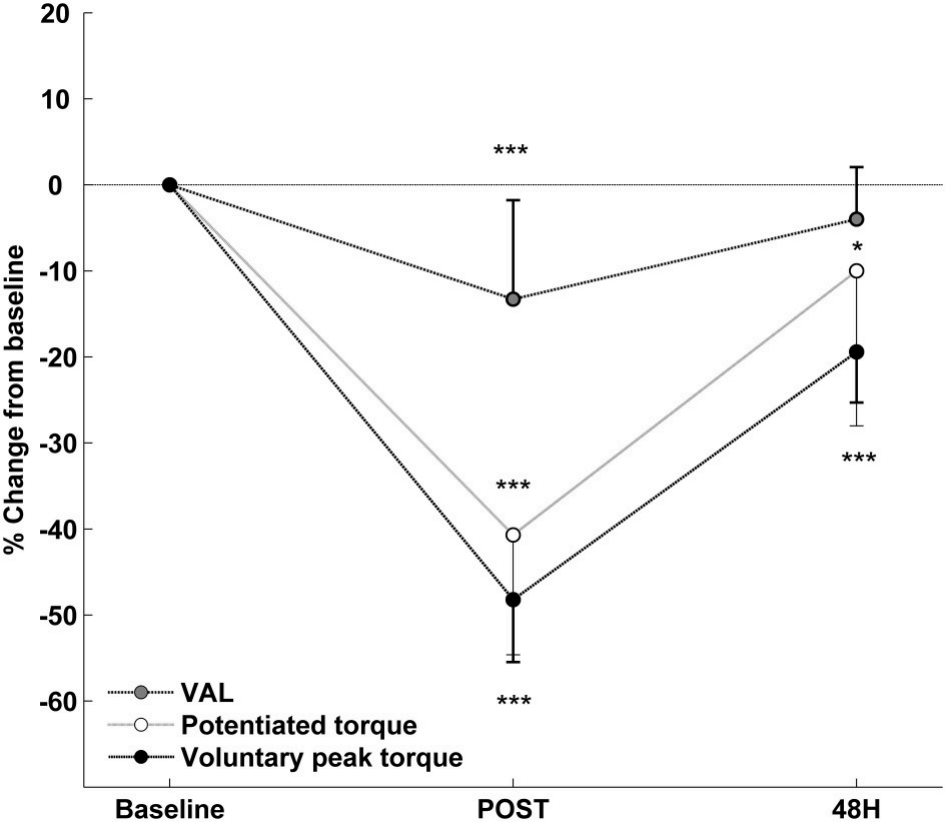
**Relative changes from baseline (mean ± SD) for voluntary isometric peak torque, potentiated evoked torque and voluntary activation level (VAL) after the exercise**. ^*^Significantly different from baseline (^*^ < 0.05; ^***^ < 0.001).

Compared with baseline, voluntary peak torque (144.2 ± 30.1 vs. 75.1 ± 21.4 N·m; *p* < 0.001, _*p*_η^2^ = 0.92), potentiated torque (44.9 ± 7.4 vs. 26.6 ± 7.2 N·m; *p* < 0.001, _*p*_η^2^ = 0.81) and VAL (99.5 ± 0.8 vs. 86.3 ± 11.9%; *p* < 0.001, _*p*_η^2^ = 0.58) significantly decreased at POST (Figure 2). At 48 h, voluntary peak torque (116.3 ± 26.6 N·m; *p* < 0.001) and potentiated torque (40.4 ± 9.5 N·m; *p* = 0.041) remained significantly lower than baseline, while VAL was not significantly different from baseline (95.5 ± 6.6%; *p* = 0.139). As shown in Table 1, the potentiated torque decrement at 48 h was positively correlated (*r* = 0.77; *p* < 0.01) with the decrease in potentiated torque at POST and negatively correlated (*r* = –0.71; *p* < 0.05) with the decrease in VAL at POST. Muscle soreness did not differ from baseline (3.2 ± 4.6 mm) at POST (12.8 ± 18.2 mm), but increased (*p* < 0.001, _*p*_η^2^ = 0.82) at 48 h (55.9 ± 19.1 mm).

**Table 1:** **Pearson's correlation coefficients (r) and *p*-values (p) for simple regressions between neuromuscular parameters measured during (CAR and lengthening torque) and after (VAL and potentiated torque) the eccentric exercise and both voluntary isometric peak torque and potentiated torque decrements at 48 h**.

**Variables**	**Voluntary peak torque decrement at 48 h**	**Potentiated torque decrement at 48 h**
**CENTRAL**		
Relative change for VAL at POST	*r* = 0.22; *p* = 0.516	*r* = −0.71; *p* = 0.014^#^
CAR averaged on the entire exercise	*r* = 0.16; *p* = 0.635	*r* = −0. 16; *p* = 0.646
CAR measured for set 1	*r* = 0.39; *p* = 0.234	*r* = 0. 09; *p* = 0.797
**PERIPHERAL**		
Relative change for potentiated torque at POST	*r* = −0.09; *p* = 0.783	*r* = 0.77; *p* = 0.005^#^
Lengthening torque averaged on the entire exercise	*r* = −0.44; *p* = 0.171	*r* = 0.55; *p* = 0.081
Lengthening torque for set 1	*r* = −0.52; *p* = 0.102	*r* = 0. 39; *p* = 0.237

# *Significant correlations (p < 0.05)*.

### Eccentric exercise

**Figure 3:**
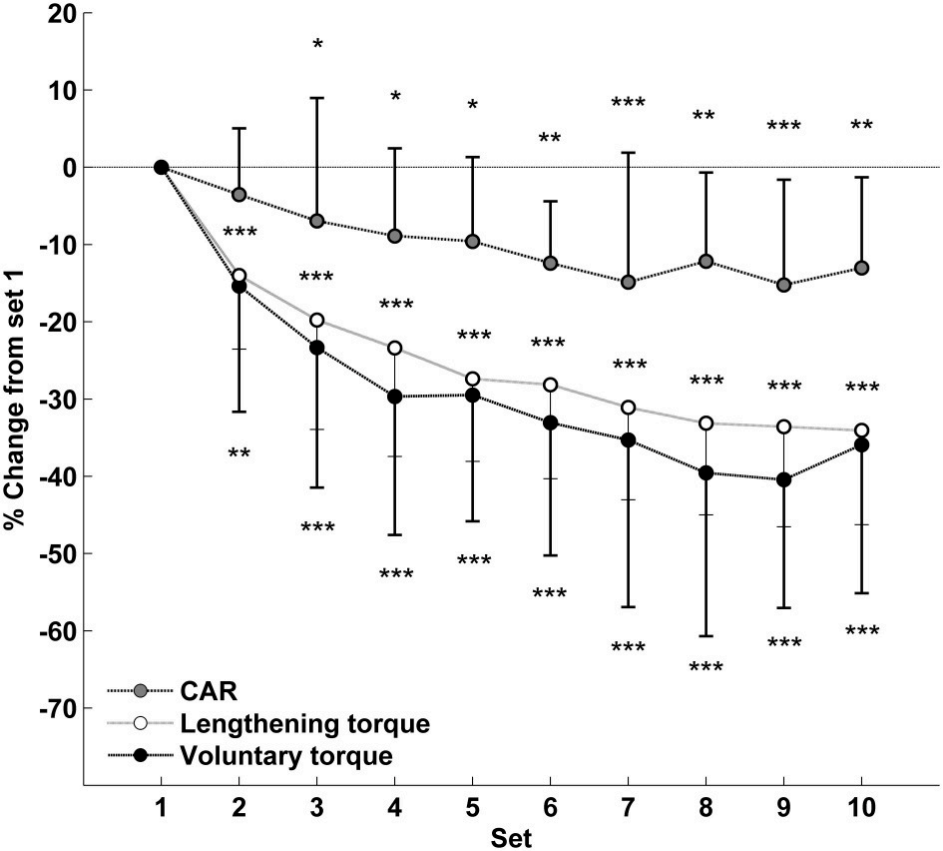
**Relative changes from set 1 (mean ± SD) for mean voluntary torque, lengthening evoked torque and central activation ratio (CAR) during the eccentric exercise**. ^*^Significantly different from set 1 (^*^ < 0.05; ^**^ < 0.01; ^***^ < 0.001).

Compared with the first set, the mean voluntary torque was significantly reduced from the second (89.1 ± 22.5 vs. 74.8 ± 18.9 N·m; *p* < 0.01; _*p*_η^2^ = 0.61; Figure 3) to the last set of the exercise (55.1 ± 16.0 N·m; *p* < 0.001). Both lengthening torque (Set 1: 1.30 ± 0.28 vs. Set 10: 0.86 ± 0.23 a.u.; *p* < 0.001; _*p*_η^2^ = 0.73) and CAR (Set 1: 87.4 ± 7.7 vs. Set 10: 75.7 ± 9.9%; *p* < 0.01; _*p*_η^2^ = 0.35) decreased during the eccentric contractions, with a significant drop from the second and the third set, respectively (Figure 3). Lengthening torque and CAR, either measured for the first set or averaged on the entire exercise, were not significantly correlated with muscle damage markers at 48 h (Table 1).

## Discussion

Our results showed a progressive decrease in voluntary torque concomitantly to a significant impairment of both central (voluntary activation) and peripheral (evoked torque) components during eccentric contractions. The long-lasting decrease in muscle strength, measured 2 days after the exercise, was related to changes in both components recorded immediately after the exercise, with no relation with either central or peripheral factors measured during the exercise.

Eccentric contractions induced a reduction in the mean voluntary torque (up to −36%) from the first to the last set. This drop in force-generating capacity was associated with both central and peripheral impairments, as reflected by the concomitant decrease in central activation ratio (up to −13%) and lengthening torque (up to −34%) during the eccentric contractions. The voluntary isometric peak torque also significantly decreased immediately after the eccentric contractions (−48%) and remained impaired 2 days later (−19%). In accordance with Paulsen et al. (2012), such early (approximately −50%) and long-lasting (approximately −20%) reductions in force-generating capacity could reflect histological disruptions classified as “moderate” exercise-induced muscle damage. At the peripheral level, the significant impairment in potentiated torque measured 2 days after the exercise (−10%) also indicated the presence of damage in the exercised muscles. Prasartwuth et al. (2006) stated that monitoring the evoked torque instead of voluntary strength after eccentric contractions could better reflect muscle recovery. However, while long-lasting alterations of muscle function could be confidently associated with the presence of muscle damage, the drop in force-generating capacity measured during and immediately after the eccentric contractions could be affected by both muscle damage and fatigue (Faulkner et al., 1993).

Excitation-contraction coupling failure has been suggested as a major mechanism responsible for damage-induced peripheral impairments (Warren et al., 2002). Despite the lower energetic cost associated with eccentric contractions compared with concentric contractions (LaStayo et al., 2014), it is likely that the 300 repeated contractions used in the present study also resulted in metabolic disturbances, representative of neuromuscular fatigue (Pasquet et al., 2000). Further, while muscle soreness and voluntary activation have been shown to behave differently after eccentric contractions (Prasartwuth et al., 2005; Behrens et al., 2012), it is still unclear how nociceptive sensory volleys triggered with the accumulation of biochemical metabolites in the damaged muscle affect neural modulations (Gandevia, 2001; Racinais et al., 2008). Therefore, muscle damage and fatigue processes may involve composite alterations in some components of the muscle function (e.g., excitation-contraction coupling, spinal excitability), and the observed neuromuscular changes must be interpreted with caution.

Stretch amplitude (Nosaka and Sakamoto, 2001), maximum force during an active stretch (Warren et al., 1993), number of eccentric actions (Warren et al., 1993), or training status (Newham et al., 1987) have been suggested as potential determinant factors in magnitude of exercise-induced muscle damage. In a previous study, Hubal et al. (2007) showed that the larger the voluntary strength loss immediately after eccentric contractions, the larger evoked torque decrements, without differences in central activation ratio between low and high responders to muscle damage. These authors concluded that the variability in strength loss after eccentric contractions was mainly driven at the muscle level. In the present study, we found a strong positive correlation between early (POST) and long-lasting (48 h) potentiated torque decrements (*r* = 0.77; *p* < 0.01). This result suggests that the muscular impairments observed in the days following the eccentric exercise were related to the immediate peripheral disruptions. On the basis of observations inferred from the mouse model, Warren et al. (2002) stated that the early force loss after eccentric contractions mainly results from excitation-contraction coupling failure (~75%) and cytoskeletal alterations (~25%), which both persist 24 h after the damaging exercise. Therefore, most of the peripheral determinants of the early strength loss were incompletely recovered in the days following eccentric contractions (Morgan and Allen, 1999; Warren et al., 2002). This feature could partly explain the strong relationship observed between potentiated torque decrements at POST and 48 h in the present study.

Central and peripheral modulations were assessed during the eccentric contractions in an attempt to better identify the relationship between the very early (i.e., from the first contractions) eccentric exercise-induced neuromuscular responses and muscle damage symptoms. Our results showed that the decreases in voluntary peak torque and potentiated torque at 48 h were not related to the lengthening torque, either assessed after the first set or averaged over the entire eccentric exercise (Table 1). Contrary to the initial hypothesis, and despite the close relationship between peripheral function and muscle damage depicted above, peripheral changes observed during the eccentric contractions were not related to the progressive apparition of muscle damage.

In the present study, we found a lower voluntary activation at the onset of the eccentric contractions (~87%) than for the baseline isometric measurement (~99%). The non-maximal neural activity observed during eccentric contractions has been mainly explained by a facilitation of spinal inhibitory pathways, despite an extra excitatory descending drive from the motor cortex (Gruber et al., 2009). Previous authors suggested that the distinctive motor output involved in eccentric contractions could act as a tension-limiting phenomenon protecting against muscle damage (Westing et al., 1991). However, this specific neural feature has never been directly compared with the magnitude of muscle damage. The present study is the first to appraise the central contribution to eccentric exercise-induced muscle damage by assessing voluntary activation during the eccentric contractions. Contrary to the initial hypothesis, our findings did not underline any relationship between muscle damage symptoms and central activation ratio, either measured during the first set or over the entire eccentric exercise. Therefore, the central nervous system should only have a limited influence on the reduction in muscle function after eccentric contractions (Pasquet et al., 2000; Hubal et al., 2007). However, it must be reminded that the assessment of voluntary activation during the eccentric exercise might represent continuous regulatory mechanisms (e.g., facilitation of inhibitory spinal pathways) associated with central fatigue, which could discredit a real tension-limiting phenomenon.

The reduction in voluntary activation measured immediately after the exercise (POST) constitutes a widespread indicator of central fatigue (Gandevia, 2001). Racinais et al. (2008) reported a decrease in voluntary activation up to 2 days after a 30-min backward downhill walking exercise. This long-lasting decrement in neural activity was suggested to be driven by nociceptive afferents associated with muscle soreness. In the present study, we did not find any alteration of the voluntary activation level 2 days after the exercise, despite a significant increase in muscle soreness. Therefore, as suggested in previous works (Prasartwuth et al., 2005; Behrens et al., 2012), the neural alterations would be only perceptible in a short-term after eccentric contractions, and would be mainly related to fatigue. However, in contrast to Hubal et al. (2007), we found a negative relationship (*r* = −0.71; *p* < 0.05) between the potentiated torque decrement 2 days after damaging exercise, indicator of muscle damage (Prasartwuth et al., 2006), and the early impairment in voluntary activation. In other words, the higher the central alterations after the exercise, the lower the long-lasting strength loss at the muscle level. Therefore, even after a maximal eccentric exercise, a muscle wisdom phenomenon arising from central components would attest for a low magnitude in long-lasting muscle function impairment. However, additional investigations, including comparison with concentric and/or isometric contractions, are needed to inspect the specific origin of central alterations with repeated eccentric contractions.

To summarize, the present study indicated that the progressive decrease in voluntary torque during eccentric contractions of plantar flexors was concomitant to an impairment of both central (voluntary activation) and peripheral (evoked torque) components. The assessment of voluntary activation during eccentric contractions did not underline any influence of the central nervous system to prevent muscle damage. However, the immediate reduction in both central and peripheral components after the eccentric contractions could reflect the magnitude of long-lasting muscle function impairments. Further investigations are needed to discriminate the contribution of muscle damage and fatigue phenomenon in the neuromuscular responses observed in the early phase after and during the eccentric exercise, by investigating central and peripheral components during other contraction modes (e.g., concentric and isometric).

## Author contributions

VD, MJ, SD, AC, LL, AG, and GG: conception and design. VD, MJ, SD, AC, LL, AG, and GG: acquisition, analysis or interpretation of data. VD, MJ, SD, AC, LL, AG, and GG: drafting and revising the article. VD, MJ, SD, AC, LL, AG, and GG: final approval of the version to be published.

### Conflict of interest statement

The authors declare that the research was conducted in the absence of any commercial or financial relationships that could be construed as a potential conflict of interest.

## References

[B1] Armstrong R. B. (1984). Mechanisms of exercise-induced delayed onset muscular soreness: a brief review. Med. Sci. Sports Exerc. 16, 529–538. 10.1249/00005768-198412000-00002 6392811

[B2] Babault N. Pousson M. Ballay Y. Van Hoecke J. (2001). Activation of human quadriceps femoris during isometric, concentric, and eccentric contractions. J. Appl. Physiol. (1985) 91, 2628–2634. 11717228 10.1152/jappl.2001.91.6.2628

[B3] Behrens M. Mau-Moeller A. Bruhn S. (2012). Effect of exercise-induced muscle damage on neuromuscular function of the quadriceps muscle. Int. J. Sports Med. 33, 600–606. 10.1055/s-0032-1304642 22510801

[B4] Cohen J. (1988). Statistical Power Analysis for the Behavioral Sciences. Hillsdale, NJ: L. Erlbaum Associates.

[B5] Dello Iacono A. Padulo J. Ayalon M. (2016). Core stability training on lower limb balance strength. J. Sports Sci. 34, 671–678. 10.1080/02640414.2015.1068437 26177151

[B6] Duchateau J. Baudry S. (2014). Insights into the neural control of eccentric contractions. J. Appl. Physiol. (1985) 116, 1418–1425. 10.1152/japplphysiol.00002.2013 23429873

[B7] Enoka R. M. (1996). Eccentric contractions require unique activation strategies by the nervous system. J. Appl. Physiol. (1985) 81, 2339–2346. 9018476 10.1152/jappl.1996.81.6.2339

[B8] Faulkner J. A. Brooks S. V. Opiteck J. A. (1993). Injury to skeletal muscle fibers during contractions: conditions of occurrence and prevention. Phys. Ther. 73, 911–921. 8248299 10.1093/ptj/73.12.911

[B9] Fridén J. Sjöström M. Ekblom B. (1983). Myofibrillar damage following intense eccentric exercise in man. Int. J. Sports Med. 4, 170–176. 10.1055/s-2008-1026030 6629599

[B10] Gandevia S. C. (2001). Spinal and supraspinal factors in human muscle fatigue. Physiol. Rev. 81, 1725–1789. 11581501 10.1152/physrev.2001.81.4.1725

[B11] Gruber M. Linnamo V. Strojnik V. Rantalainen T. Avela J. (2009). Excitability at the motoneuron pool and motor cortex is specifically modulated in lengthening compared to isometric contractions. J. Neurophysiol. 101, 2030–2040. 10.1152/jn.91104.2008 19193768

[B12] Guilhem G. Cornu C. Nordez A. Guével A. (2010). A new device to study isoload eccentric exercise. J. Strength Cond. Res. 24, 3476–3483. 10.1519/JSC.0b013e3181d640ec 20733524

[B13] Herzog W. (2014). Mechanisms of enhanced force production in lengthening (eccentric) muscle contractions. J. Appl. Physiol. (1985) 116, 1407–1417. 10.1152/japplphysiol.00069.2013 23429875

[B14] Howatson G. van Someren K. A. (2007). Evidence of a contralateral repeated bout effect after maximal eccentric contractions. Eur. J. Appl. Physiol. 101, 207–214. 10.1007/s00421-007-0489-5 17534644

[B15] Hubal M. J. Rubinstein S. R. Clarkson P. M. (2007). Mechanisms of variability in strength loss after muscle-lengthening actions. Med. Sci. Sports Exerc. 39, 461–468. 10.1249/01.mss.0000247007.19127.da 17473772

[B16] Kellis E. Baltzopoulos V. (1995). Isokinetic eccentric exercise. Sports Med. 19, 202–222. 10.2165/00007256-199519030-00005 7784759

[B17] LaStayo P. Marcus R. Dibble L. Frajacomo F. Lindstedt S. (2014). Eccentric exercise in rehabilitation: safety, feasibility, and application. J. Appl. Physiol. (1985) 116, 1426–1434. 10.1152/japplphysiol.00008.2013 23823152

[B18] Leys C. Ley C. Klein O. Bernard P. Licata L. (2013). Detecting outliers: Do not use standard deviation around the mean, use absolute deviation around the median. J. Exp. Soc. Psychol. 49, 764–766. 10.1016/j.jesp.2013.03.013

[B19] Mair J. Koller A. Artner-Dworzak E. Haid C. Wicke K. Judmaier W.. (1992). Effects of exercise on plasma myosin heavy chain fragments and MRI of skeletal muscle. J. Appl. Physiol. (1985) 72, 656–663. 1559945 10.1152/jappl.1992.72.2.656

[B20] McHugh M. P. (2003). Recent advances in the understanding of the repeated bout effect: the protective effect against muscle damage from a single bout of eccentric exercise. Scand J. Med. Sci. Sports 13, 88–97. 10.1034/j.1600-0838.2003.02477.x 12641640

[B21] Miller J. (1991). Reaction time analysis with outlier exclusion: bias varies with sample size. Q. J. Exp. Psychol. A 43, 907–912. 10.1080/14640749108400962 1775668

[B22] Morgan D. L. Allen D. G. (1999). Early events in stretch-induced muscle damage. J. Appl. Physiol. 87, 2007–2015. 10601142 10.1152/jappl.1999.87.6.2007

[B23] Moritani T. Muramatsu S. Muro M. (1987). Activity of motor units during concentric and eccentric contractions. Am. J. Phys. Med. 66, 338–350. 3324772

[B24] Newham D. J. Jones D. A. Clarkson P. M. (1987). Repeated high-force eccentric exercise: effects on muscle pain and damage. J. Appl. Physiol. (1985) 63, 1381–1386. 3693172 10.1152/jappl.1987.63.4.1381

[B25] Newham D. J. Mills K. R. Quigley B. M. Edwards R. H. (1983). Pain and fatigue after concentric and eccentric muscle contractions. Clin. Sci. 64, 55–62. 10.1042/cs0640055 6822050

[B26] Nosaka K. Sakamoto K. (2001). Effect of elbow joint angle on the magnitude of muscle damage to the elbow flexors. Med. Sci. Sports Exerc. 33, 22–29. 10.1097/00005768-200101000-00005 11194107

[B27] Pasquet B. Carpentier A. Duchateau J. Hainaut K. (2000). Muscle fatigue during concentric and eccentric contractions. Muscle Nerve 23, 1727–1735. 11054752 10.1002/1097-4598(200011)23:11<1727::aid-mus9>3.0.co;2-y

[B28] Paulsen G. Mikkelsen U. R. Raastad T. Peake J. M. (2012). Leucocytes, cytokines and satellite cells: what role do they play in muscle damage and regeneration following eccentric exercise? Exerc Immunol Rev 18, 42–97. 22876722

[B29] Place N. Maffiuletti N. A. Martin A. Lepers R. (2007). Assessment of the reliability of central and peripheral fatigue after sustained maximal voluntary contraction of the quadriceps muscle. Muscle Nerve 35, 486–495. 10.1002/mus.20714 17221875

[B30] Prasartwuth O. Allen T. J. Butler J. E. Gandevia S. C. Taylor J. L. (2006). Length-dependent changes in voluntary activation, maximum voluntary torque and twitch responses after eccentric damage in humans. J. Physiol. 571, 243–252. 10.1113/jphysiol.2005.101600 16357013 PMC1805656

[B31] Prasartwuth O. Taylor J. L. Gandevia S. C. (2005). Maximal force, voluntary activation and muscle soreness after eccentric damage to human elbow flexor muscles. J. Physiol. 567, 337–348. 10.1113/jphysiol.2005.087767 15946963 PMC1474152

[B32] Racinais S. Bringard A. Puchaux K. Noakes T. D. Perrey S. (2008). Modulation in voluntary neural drive in relation to muscle soreness. Eur. J. Appl. Physiol. 102, 439–446. 10.1007/s00421-007-0604-7 17978834 PMC2267484

[B33] Starbuck C. Eston R. G. (2012). Exercise-induced muscle damage and the repeated bout effect: evidence for cross transfer. Eur. J. Appl. Physiol. 112, 1005–1013. 10.1007/s00421-011-2053-6 21720885

[B34] Warren G. L. Hayes D. A. Lowe D. A. Armstrong R. B. (1993). Mechanical factors in the initiation of eccentric contraction-induced injury in rat soleus muscle. J. Physiol. (Lond). 464, 457–475. 10.1113/jphysiol.1993.sp019645 8229813 PMC1175396

[B35] Warren G. L. Hermann K. M. Ingalls C. P. Masselli M. R. Armstrong R. B. (2000). Decreased EMG median frequency during a second bout of eccentric contractions. Med. Sci. Sports Exerc. 32, 820–829. 10.1097/00005768-200004000-00015 10776902

[B36] Warren G. L. Ingalls C. P. Lowe D. A. Armstrong R. B. (2002). What mechanisms contribute to the strength loss that occurs during and in the recovery from skeletal muscle injury? J. Orthop. Sports Phys. Ther. 32, 58–64. 10.2519/jospt.2002.32.2.58 11838581

[B37] Westing S. H. Cresswell A. G. Thorstensson A. (1991). Muscle activation during maximal voluntary eccentric and concentric knee extension. Eur. J. Appl. Physiol. Occup. Physiol. 62, 104–108. 10.1007/BF00626764 2022197

